# Implications for post critical illness trial design: sub-phenotyping trajectories of functional recovery among sepsis survivors

**DOI:** 10.1186/s13054-020-03275-w

**Published:** 2020-09-25

**Authors:** Zudin A. Puthucheary, Jochen S. Gensichen, Aylin S. Cakiroglu, Richard Cashmore, Lara Edbrooke, Christoph Heintze, Konrad Neumann, Tobias Wollersheim, Linda Denehy, Konrad F. R. Schmidt

**Affiliations:** 1 William Harvey Research Institute, Barts and The London School of Medicine and Dentistry, Queen Mary University of London, London, UK; 2 Critical Care and Perioperative Medicine Research Group, Adult Critical Care Unit, Royal London Hospital, London, E1 1BB UK; 3 Institute of General Practice and Family Medicine, Jena University Hospital, Jena, Germany; 4 Institute of Family Medicine, University Hospital of the Ludwig Maximilian University, Munich, Germany; 5 Center of Sepsis Care and Control, Jena University Hospital, Jena, Germany; 6 The Francis Crick Institute, London, UK; 7 Physiotherapy Department, The University of Melbourne, Melbourne, Australia; 8 Allied Health Department, Peter MacCallum Cancer Centre, Melbourne, Australia; 9 Institute of General Practice and Family Medicine, Charité University Medicine Berlin, Berlin, Germany; 10 Institute of Biometry and Clinical Epidemiology, Charité University Medicine Berlin, Berlin, Germany; 11 Department of Anesthesiology and Operative Intensive Care Medicine, Charité University Medicine Berlin, corporate member of Freie Universität Berlin, Humboldt Universität zu Berlin, and Berlin Institute of Health, Berlin, Germany

**Keywords:** Sepsis, Post intensive care syndrome (PICS), Physical function, Health-related quality of life (HRQoL), Patient-reported outcome measures (PROMS), Co-morbidity

## Abstract

**Background:**

Patients who survive critical illness suffer from a significant physical disability. The impact of rehabilitation strategies on health-related quality of life is inconsistent, with population heterogeneity cited as one potential confounder. This secondary analysis aimed to (1) examine trajectories of functional recovery in critically ill patients to delineate sub-phenotypes and (2) to assess differences between these cohorts in both clinical characteristics and clinimetric properties of physical function assessment tools.

**Methods:**

Two hundred ninety-one adult sepsis survivors were followed-up for 24 months by telephone interviews. Physical function was assessed using the Physical Component Score (PCS) of the Short Form-36 Questionnaire (SF-36) and Activities of Daily Living and the Extra Short Musculoskeletal Function Assessment (XSFMA-F/B). Longitudinal trajectories were clustered by factor analysis. Logistical regression analyses were applied to patient characteristics potentially determining cluster allocation. Responsiveness, floor and ceiling effects and concurrent validity were assessed within clusters.

**Results:**

One hundred fifty-nine patients completed 24 months of follow-up, presenting overall low PCS scores. Two distinct sub-cohorts were identified, exhibiting complete recovery or persistent impairment. A third sub-cohort could not be classified into either trajectory. Age, education level and number of co-morbidities were independent determinants of poor recovery (AUROC 0.743 ((95%CI 0.659–0.826), *p* < 0.001). Those with complete recovery trajectories demonstrated high levels of ceiling effects in physical function (PF) (15%), role physical (RP) (45%) and body pain (BP) (57%) domains of the SF-36. Those with persistent impairment demonstrated high levels of floor effects in the same domains: PF (21%), RP (71%) and BP (12%). The PF domain demonstrated high responsiveness between ICU discharge and at 6 months and was predictive of a persistent impairment trajectory (AUROC 0.859 (95%CI 0.804–0.914), *p* < 0.001).

**Conclusions:**

Within sepsis survivors, two distinct recovery trajectories of physical recovery were demonstrated. Older patients with more co-morbidities and lower educational achievements were more likely to have a persistent physical impairment trajectory.

In regard to trajectory prediction, the PF score of the SF-36 was more responsive than the PCS and could be considered for primary outcomes. Future trials should consider adaptive trial designs that can deal with non-responders or sub-cohort specific outcome measures more effectively.

## Background

Increasing numbers of patients are successfully surviving critical illness. Unfortunately, residual functional and/or mental disabilities affect many critical care survivors after hospital discharge [1, 2]. Despite extensive research into rehabilitation strategies, few studies have been able to demonstrate a positive effect on this ensuing dysfunction or improve health-related quality of life (HRQoL) [3–6]. Given that rehabilitation strategies have a strong evidence base in other patient populations [7], trial-related methodological issues have been proposed as a source of influence in this area and examined [8, 9].

Population heterogeneity within the critically ill cohort is one area that may hinder current outcome analysis. Certain specific patient characteristics have already been identified as influential in regard to an individuals’ subsequent HRQoL outcome. To date, these include age [10], pre-critical illness comorbidity [11] and socioeconomic status [12]. Severity of critical illness, intensive care unit (ICU) length of stay and the effect of within-ICU physiology remain unclear influences, as does sex [10, 11, 13–16]. If these factors are not accounted for in a trial design, patient stratification, or analysis, outcome data may be unintentionally skewed. Many of the current outcome assessments for trials in critical care fail to account for these confounders [15, 17]. Patient-reported outcome measures are increasingly prioritised as endpoints [18–20]. The Physical Component Score (PCS) of the Short Form-36 Questionnaire (SF-36) is used to demonstrate the physical disability of critical care survivors [21] and is widely reported in rehabilitation trials.

Several re-analyses have demonstrated sub-phenotypes based on recovery trajectories [9, 15, 22]. How these sub-phenotypes respond to the variety of assessments that measure HRQoL currently in use is not yet defined. It may be that these assessments, often applied as outcome measures, have different clinimetric properties within patient sub-populations. Understanding this aspect of measurement in addition to recovery trajectories will be important to future trial design and outcome interpretation.

We performed a secondary analysis of a critical care trial of sepsis survivors using 2-year follow-up data [23]. The aim of this was to (i) examine the trajectories of functional recovery in critically ill patients using an agnostic approach to delineate patient sub-phenotypes; (ii) examine the distinguishing clinical characteristics between these cohorts and (iii) assess the differences in clinimetric properties of assessment tools of physical function between cohorts.

## Methods

The patient cohort comprised of those recruited to a randomised control trial conducted between February 2011 and December 2015 evaluating a primary care-based sepsis aftercare intervention [23, 24]. Two hundred ninety-one adult survivors of sepsis were recruited from nine centres across Germany. Trial design, methodology and outcomes are described in detail in the original manuscript [23, 25]. Briefly, trained study nurses collected baseline data at in-person interviews while participants were still hospitalised. Follow-up data pertaining to HRQoL and physical function were collected at 6 months, 12 months and 24 months by telephone interviews. Those instruments specific to this analysis were the Physical Component Score (PCS) of the SF-36 [26], three of its four subdomains (physical function, role physical and body pain), activities of daily living (ADL) and the Extra Short Musculoskeletal Function Assessment regarding physical function and disability (XSFMA-F/B) [27]. This extra short questionnaire is derived from the 101-item Musculoskeletal Function Assessment (MFA) by Engelberg et al. to assess functional status from the patient’s perspective [28]. It has been mainly used in Germany for patients following orthopaedic surgery [27]. Functional outcome data were also analysed for sub-phenotype concurrent validity and clinimetric properties. Both randomisation groups were included into analyses, as no effects of the intervention were shown regarding functional or HRQoL outcomes [23]. Only those with complete data sets (all four time points) were used in this analysis.

Education and family status classifications are shown in Additional Table 1 and addressed domains of instruments used in Additional Table 1.1.

### Trajectory projection cluster analysis

Groups of longitudinal trajectories of Physical Component Scores of the SF-36 (the most commonly reported 6-month HRQoL outcome measure [3, 6, 29–34]) were clustered using the R-package TRAJ [35–37] and applied. Briefly, this package implements a 3-step procedure [36]. Firstly, 24 summary measures (available in Additional Table 2) are calculated that measure the features of trajectories. These measures were then analysed using factor analysis to select those that best describe the main features of trajectories. Lastly, using these factors the trajectories were clustered.

### General statistical analysis

Continuous data were assessed for normality using D’Agostino and Pearson omnibus normality tests and analysed using paired two-tailed Student’s *t* test or Mann-Whitney *U* test as appropriate. Normally distributed data were described using the mean (95% confidence interval) and non-normally distributed data as median (interquartile range). Categorical variables were analysed by *χ*^2^ testing. Multivariable and univariable logistic regression analyses were applied to variables potentially determining cluster allocation (dependent variable). Unclustered participants were not used in the logistical analysis, and a multinomial regression performed as a sensitivity analysis. Independent variables were determined as characteristics (Table 1), with a univariable screening threshold set at *p* < 0.10. Significance for all other tests was set at *p* < 0.05. The area under the receiver-operator-curve was used to test the predictive capacity of early ICU discharge and 6 months of assessments for persistent functional impairment.

**Table 1:** Baseline characteristics of different cohorts

	Persistent impairment	NA	Complete recovery	NA	Unclustered	NA	
*n*	76		61		22		
Age (years)	65 (54.3–72)		56 (43–70)		63 (52–69.3)		***p*** = 0.002*
Male sex (*n*)^#^	47 (61.8%)		44 (72.1%)		16 (72.7%)		*p* = 0.205
ICULOS	23.0 (12.8–39.5)	2	19 (10.0–31.0)	6	40.5 (15.3–48.3)	2	*p* = 0.207
MV(day)	9 (2–20)	1	6 (2–22)	2	10 (4–29)	3	*p* = 0.746
CCI	3 (1–5.8)		3 (1–5)	1	2.5 (1.8–6)		*p* = 0.246
RRT (day)	0 (0–0.75)		0 (0–2.5)	3	0 (0–2.5)		*p* = 0.650
Tracheostomy (*n*)^#^	20 (26.3%)	21	18 (29.5%)	13	11 (50%)	3	*p* = 0.678
Intervention group (*n*)^#^	38 (50%)		38 (62.2%)		11(50%)		*p* = 0.150
Education^ǂ$^	5 (1–9)		5 (2–9)		5 (2–9)		***p*** = 0.039*
BMI	27.8 (24.4–32.5)		25.8 (22.6–29.1)	1	26.7 (23–30)	2	***p*** = 0.006*
Family status^ǂ$^	2 (1–6)	1	2(1–6)		2(1–4)	1	***p*** = 0.021*
No. of ICD diagnoses at discharge	9 (6–15)		9 (5–11)		8 (6–15.8)		*p* = 0.077

Data are shown as medians (interquartile ranges), except for percentages and mode (range). *p* values represent Mann-Whitney *U* tests between persistent impairment and complete recovery, except for ^#^chi-squared test

*ICULOS* intensive care length of stay (days), *MV*(d) period of mechanical ventilation (days), *CCI* Charlston Co-morbidity Index, *RRT*(d) renal replacement therapy (days) and *NA* not available

^$^Indicated mode (range) with the significance taken to be *p* < 0.05

*p < 0.05

^ǂ^Categories shown in Additional Table 1

### Floor and ceiling effects

Scores at their lowest point are defined as ‘floor effects’ and a ‘ceiling effect’ occurs where patients ‘may show no improvement in function if a functional scale is not able to assess high-level instrumental ADLs (a ceiling effect) [38, 39]. Floor and ceiling effects render a measure unable to discriminate between participants at either extreme of the scale. This negatively affects measurement properties, including sample size requirements. Reducing these effects by choice of the right measure can therefore improve study efficiency [40]. Floor effects were calculated as the percentage of participants scoring the worst possible score for the measure. Ceiling effects were calculated as the percentage of participants scoring the best possible score for the measure. Components of the SF-36 were examined at the differing time points for floor and ceiling effects, for the cohort as a whole and for the individual clusters. Floor and ceiling effects were considered relevant if > 15% of the participants had the highest or lowest score respectively [41].

### Concurrent validity

Concurrent validity is a measure of how well a test compares to a gold standard (such as the PCS) [38] and its substitutability. Therefore, it is a component of criterion validity, an estimate of accuracy based on an external criterion [42]. Coefficient of determination from regression between parameters was used to measure concurrent validity (the degree to which a test can be used as a substitute measure for the gold standard) between the PCS and PF of the SF-36, ADLs and XSFMA-F/B. All coefficients were interpreted as little (0.00–0.25), fair (0.25–0.50), moderate (0.50–0.75) and excellent association (0.75–1.0) [43].

### Responsiveness

Responsiveness is a measure of sensitivity to change and discriminatory properties (the ability to detect a clinically relevant change in health status over time), and part of the COSMIN checklist (COnsensus-based Standards for the selection of health Measurement INstruments) [42, 44, 45]. Change in scores from hospital discharge to 24 months was assessed using paired *t* tests and data represented as mean difference and 95% CI [43]. Responsiveness of each test to time/recovery post critical illness was calculated using the effect size index, calculated as the mean change score divided by the baseline pooled standard deviation [38, 46]. Changes were interpreted according to Cohen’s d effect size as small (0.2 to 0.49), moderate (0.5 to 0.79) and large (> 0.80) [47, 48].

## Results

Of the original 291 participants recruited, 24-month follow-up data was collected on 186 participants (41 lost to follow-up, 64 died < 24 months). Complete data was available on 159 participants who were included in the final analyses. Those with incomplete follow-up were not included. When compared, those who died were older, had a longer length of stay and more co-morbidities, all of which is not unexpected (see Additional Table 3).

**Fig. 1:**
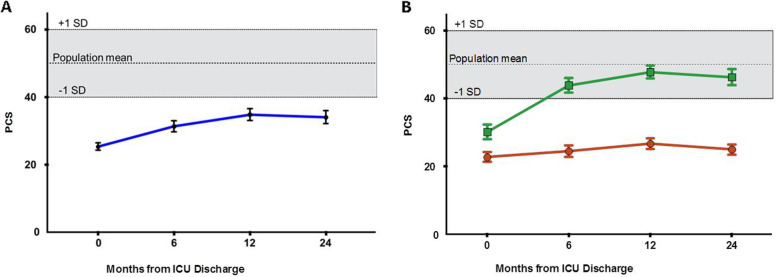
Trajectory of physical recovery over 24 months. Indicated by the Physical Component Score (PCS) of the SF-36, mean (95%CI) of. **a** All patients and **b** two sub-cohorts: green line: complete recovery, red line: persistent impairment *represents *p* < 0.05 for unpaired two-tailed Student’s *T* tests. Dotted line represents population norms

PCS of the SF-36 for critically ill participants were reduced relative to population norms at ICU discharge and remained low at 24 months (Fig. 1a).

### Trajectory clustering

Trajectory projection analysis identified two distinct sub-cohorts: one cohort exhibited a faster and more complete recovery trajectory defined as within one standard deviation of population norms (*n* = 61). A second cohort exhibited more persistent functional impairment (*n* = 76) (Fig. 1b). The remaining 22 participants were not classified into either cohort, as no clear trajectory was seen (Additional Fig. 2). The differing characteristics of the cohorts are shown in Table 1.

**Fig. 2:**
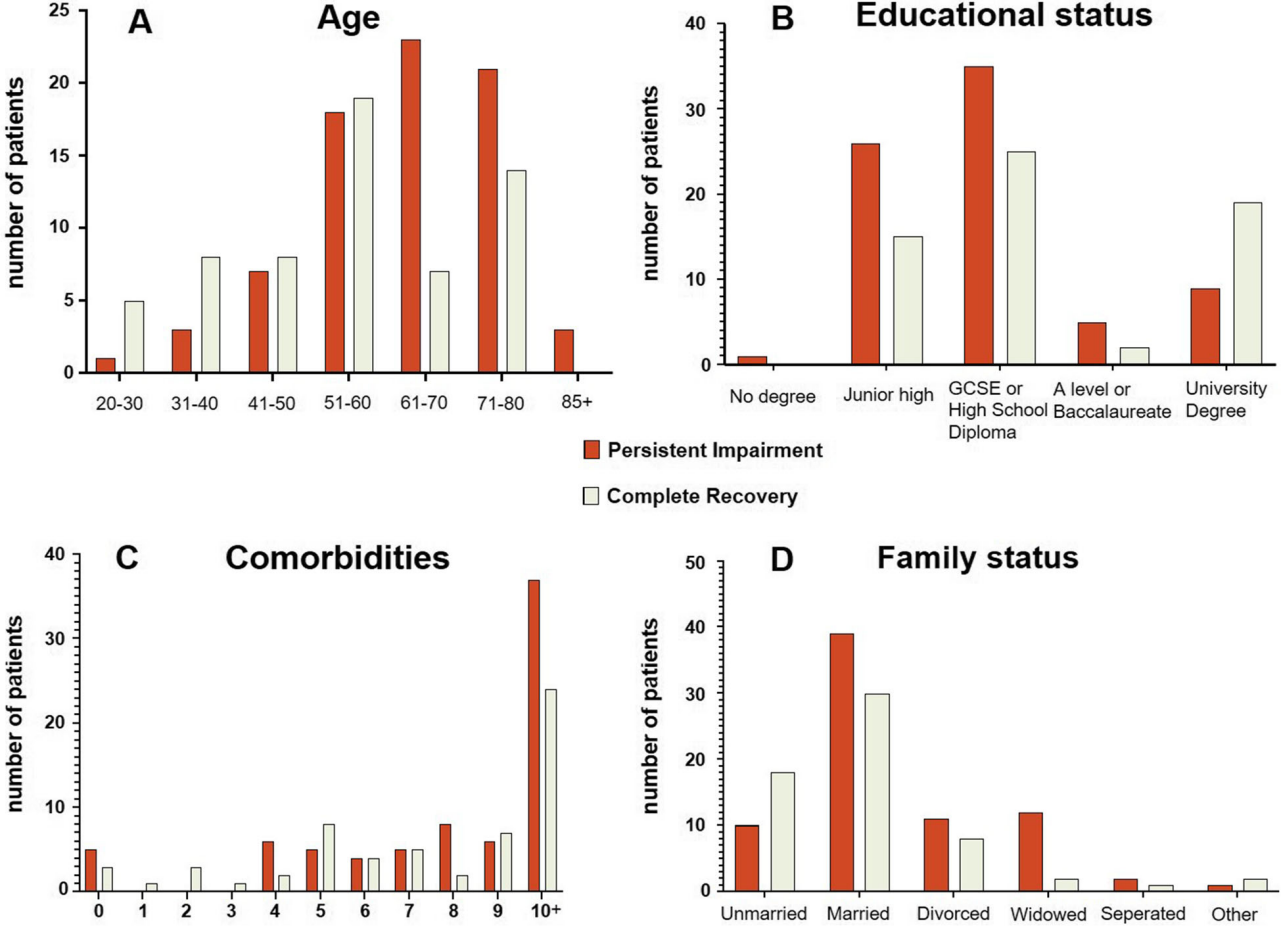
Distribution of characteristics of both cohorts. For each figure, red columns represent the persistent impairment cohort, green columns represent the complete recovery cohort, broken down by **a** age, **b** education status, **c** number of co-morbidities and **d** family status

The complete recovery cohort was on average younger (56 years (IQR 43–70) vs. 65 years (IQR 54–72), *p* = 0.002, Fig. 2a), with higher education levels (5(4–8) vs. 5(3–5), *p* = 0.039, Fig. 2b), more likely to be unmarried (Fig. 2d) and had a lower BMI (25.8(22–29) vs. 27.8(24–32), *p* = 0.006.

A multivariable logistic regression analysis demonstrated age, education level and number of co-morbidities as independent determinants of poor recovery (Additional Table 4). A model with these factors had a predictive capacity with an AUROC of 0.743 ((95%CI 0.659–0.826); *p* < 0.001; Additional Fig. 1) for cohort membership and was not over-fitted (Hosmer-Lemeshow statistic 8.456, *p* = 0.390). Neither body mass index nor family status at discharge were significant within this analysis. In a multinomial analysis, age and education remained independent determinants of recovery with the addition of body mass index (Additional Table 4.1) but not the number of co-morbidities (*p* = 0.051). No determinants were independently associated with the unclustered trajectory (see Additional Table 4.2).

### Floor and ceiling effects

**Fig. 3:**
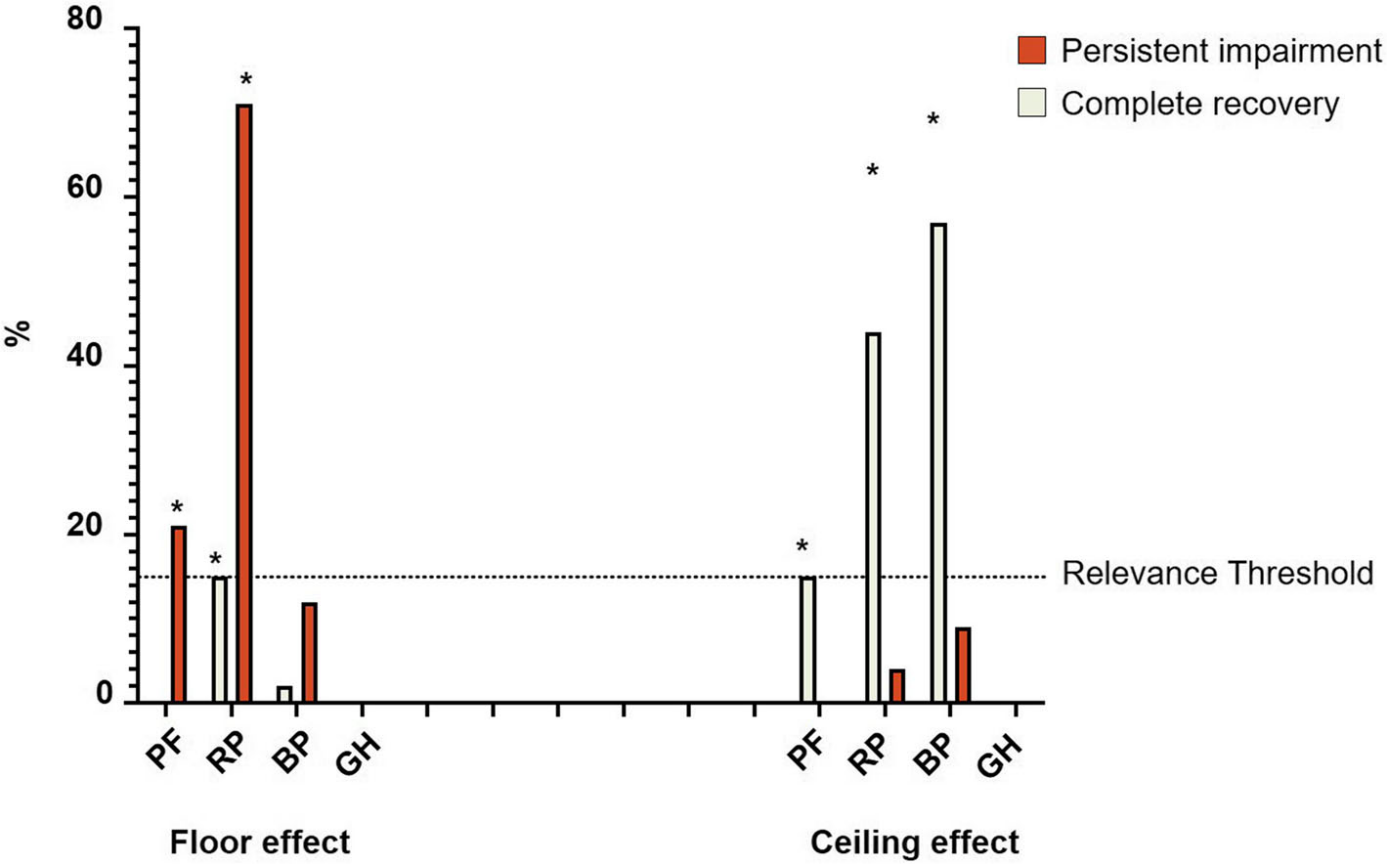
SF-36 components floor and ceiling effects. Red columns represent the persistent impairment cohort, and green the completed recovery cohort, both at 24 months. PF physical function, RP role physical, BP bodily pain and GH general health. *A value of > 15% denoting relevant effect

At a 24-month follow-up, participants in the completed recovery cohort demonstrated relevant ceiling effects within the physical function (15%), role physical (45%) and body pain (57%) domains of the SF-36. In contrast, those participants with persistent functional disability demonstrated the reverse, with relevant floor effects within physical function (21%) and role physical (71%) but not bodily pain (12%), see Table 2 and Fig. 3. These results were relatively consistent over the preceding 24 months (Additional Tables 5A and B). Floor scores at ICU discharge were only moderately associated with a persistent functional impairment trajectory (PF (AUROC 0.609 (95%CI 0.537–0.681); *p* = 0.002) and RP (AUROC 0.653 (95%CI 0.584–0.721); *p* < 0.001)). However, floor scores at 6 months were good predictors of a trajectory of persistent functional impairment (RP (AUROC 0.586 (95%CI 0.513–0.658); *p* = 0.014)), and PF (AUROC 0.938 (95%CI 0.901–0.974); *p* < 0.001)).

**Table 2:** SF-36 components floor and ceiling effects at 24 months after ICU discharge

Follow-Up	Whole cohort *N* = 159		Completed recovery *N* = 61		Persistent impairment *N* = 76	
	Floor (0)	Ceiling (100)	Floor (0)	Ceiling (100)	Floor (0)	Ceiling (100)
**PF**	16 (10)	9 (6)	0 (0)	9 (15)*	16 (21)*	0 (0)
**RP**	71 (45)*	35(22)*	9 (15)*	27 (45)*	54 (71)*	3 (4.0)
**BP**	11 (7)	52(33)*	1 (2)	35 (57)*	9 (12)	7 (9.2)
**GH**	0(0)	0(0)	0(0)	0(0)	0(0)	0(0)
**XSFMA-F**	29(18)*	0(0)	29 (46)	0(0)	0(0)	0(0)

Data are shown as numbers of patients with percentages. Data of unclustered group (*n* = 22) not shown (raw data shown in Additional Fig. 2)

*PF* physical function, *RP* role physical, *BP* bodily pain and *GH* general health, *XSFMA-F* Extra Short Form Musculoskeletal Function Assessment regarding physical function (F)

*A value of > 15% denoting relevant effects [41]

### Concurrent validity

Those participants with complete recovery demonstrated moderate to excellent concurrent validity between SF-36 PCS and both XSFMA-B AND XSFMA-F, and fair validity with ADL scores. Those participants with persistent disability demonstrated moderate concurrent validity between SF-36 PCS and both XSFMA-B AND XSFMA-F, and fair validity with ADL scores (Table 3).

**Table 3:**
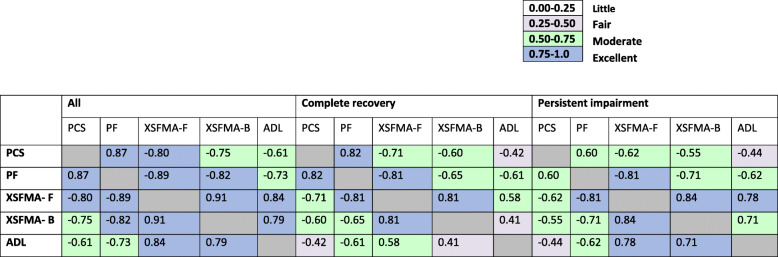
Concurrent validity of physical function assessment tools

Data shown as coefficients of determination at 24 months after ICU discharge

*PCS* Physical Component Score of the SF-36, *PF* physical function subscore, *XSFMA-F/B* Extra Short Form Musculoskeletal Function Assessment regarding physical function (F) and disability (B) and *ADL* activities of daily living

### Responsiveness

High responsiveness was seen in the complete recovery group at all time points in the Physical Component Score (> 1.0) and most notably in the physical function domain (> 1.6), with a similar pattern seen in role physical. However, this was not seen in the persistent impairment cohort, where physical function and role physical achieved only moderate responsiveness at 6 months (> 0.7). All other scores and time points demonstrated at best-limited responsiveness (Table 4). PF responsiveness between ICU discharge and 6 months was predictive of a trajectory of persistent impairment (AUROC 0.859 (95%CI 0.804–0.914); *p* < 0.001).

**Table 4:**
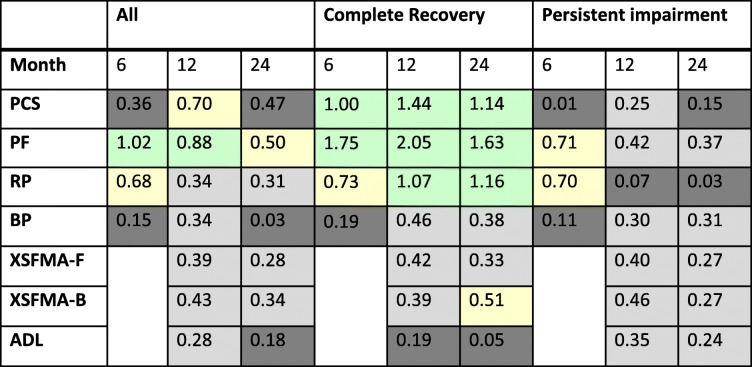
Responsiveness of physical function scores at 6, 12 and 24 months post ICU discharge

Responsiveness was measured using Cohens’ d, with changes interpreted as minimal (0.0 to 0.2, dark grey) small (0.2 to 0.49, grey), moderate (0.5 to 0.79, yellow) and large (> 0.80, green). Six-month XSFMA-F/B data were used as the baseline for responsiveness

## Discussion

This post hoc study examines the trajectories of functional impairment in cohorts of sepsis survivors regarding sub-phenotypes and specific clinical characteristics.

Two distinct sub-cohorts were identified: one of faster and more complete recovery and the other of slower recovery with more persistent functional impairment. A third sub-cohort could not be classified into either trajectory. This study also demonstrates that the older patient with more co-morbidities and with lower educational achievements is more likely to have a trajectory associated with persistent functional impairment. Importantly, the measures used exhibit very different clinimetric properties when HRQoL is measured longitudinally in different sub-cohorts. Those with good recovery have significant ceiling effects with the physical components of the SF-36 questionnaire and demonstrate high responsiveness over time. The reverse is seen in those with persistent impaired HRQoL, where significant floor effects are seen and limited responsiveness. Moderate to excellent concurrent validity was obtained across tests of HRQoL and physical function. The physical function (PF) score had the highest degrees of responsiveness across sub-cohorts and time and was predictive of a trajectory of persistent impairment when measured up to 6 months. Scoring the lowest value of PF at 6 months also was predictive of poorer outcomes at 24 months, which might be an indicator for the necessity to develop individualised rehabilitation programs for every patient.

### Individual patient characteristics

These data reiterate the role that age and multiple chronic diseases have on recovery of physical HRQoL post critical illness. Interestingly, the individual odds ratios for these factors are lower than that of educational status. This may be because educational status is reflective of poorly quantified and measured socioeconomic factors as well as individual coping abilities that are essential for the rehabilitation process [12]. However, chronological age is increasingly recognised as less accurate in terms of function relative to physiological age in the elderly [49], and the Charlston Co-morbidity Index was not designed or validated for the critical care survivor population. Ultimately, these data demonstrate that stratification (or population enrichment strategies) on one or two of these variables are unlikely to be sufficient. We have begun to understand how frailty, cognitive deficits [50], comorbidities [9], age and ICU length of stay [22, 51] interact to result in post critical illness disability, and our data confirm these findings but also suggest that these factors need to be integrated with socioeconomic data for improved identification of sub-phenotypes. The impact of social isolation is reported in other chronic diseases and needs more attention in critical illness populations [12].

### Physical function and health-related quality of life outcome measures

The use of HRQoL and patient-reported outcome measures is important and increasingly mandated, and the data reported here may help to focus the field on the appropriateness of the specific domains of the SF-36 to measure HRQoL in different subpopulations with different illness trajectories. The PCS has been used as a primary outcome measure in rehabilitation trials [6, 29], in nutrition intervention trials [52] and is in general the most commonly reported 6-month HRQoL outcome measure [3, 6, 29–34]. The PF subscore has also been used as a primary outcome measure in critical illness [53]. Fundamentally, selection of an outcome measure assumes that the intervention is suitably designed with the primary outcome in mind. When evaluating rehabilitation trials if the primary outcome of a trial is health-related quality of life, then using the summative score (PCS, incorporating all subdomains to reflect overall health-related quality of life) would be appropriate. In contrast, if the primary outcome is physical function, then it may be more appropriate to select the physical function subdomain as the measure used to evaluate the trial. It should be noted that HRQoL outcome measures have often been shown to not be sensitive enough to be affected by the biological efficacy of current post ICU interventions [54].

To date, little exploration of the most sensitive component of the SF-36 to use in trials of rehabilitation interventions has been conducted [55]. Physical and mental health factors account for 80–85% of the reliable variance in the 8 scales of the SF-36 [56]. A scoring assumption central to the summative scores (i.e., PCS and MCS) is that score aggregation could occur without score standardisation or item weighing [57]. Our data challenge this assumption: in the presence of significant heterogeneity of physical HRQoL and disability post critical illness, individual domains are more appropriate outcome measures than summative scores for physical rehabilitation trials, given the responsiveness and predictive outcomes seen across patient sub-phenotypes. Of note, the PF score has long been known to be the most valid scale for physical activity [58] and our data demonstrate that aggregating PF with the other components of the PCS decreases the clinimetric strength. The PF domain includes questions related to activities needed for daily living rather than also including return to work and questions about pain as found in the PCS. The PF domain includes several advanced mobility measures, independent activities of daily living, some activities of daily living as well as several items of the XSFMA, which may explain the concurrent validity findings, as this may be better viewed as construct validity. It may be that in the post critical illness population, there is a more specific objective perception of physical function (the PF score, comprising of 10 questions), resulting in higher responsiveness than broader subjective limitations in daily life (the RP score, comprising of 4 questions, or General Health comprising of 5 questions) or perception of pain (the BP score, comprising of 2 questions). However, the PF score also has significant ceiling effects (in those that recover) and floor effects (in those with persistent disability), suggesting the need for concurrent measurement of other more specific outcome measures such as the XSFMA-F which showed excellent validity with the SF-36 PF to address this. Notably, using the PF domain score at 6 months can predict poorer physical HRQoL outcomes and may help to guide further community or out-patient based individualised rehabilitation treatment.

### Strengths and limitations

A major strength of these analyses are the data themselves—few long-term cohort studies exist with serial contemporaneous HRQoL and physical function data to allow detailed clinimetric testing of outcome measures. The cohort size was large relative to other long-term cohort studies with serial contemporaneous HRQoL and physical function data. It is widely accepted, and accords with common sense, that the imputation of missing data on HRQoL for a deceased participant is inappropriate [59]. This is in keeping with approaches applied to randomised controlled trials [60] and is an approach used by others (with specific expertise in imputation) within the field of rehabilitation [59, 61]. This would also be consistent with analyses applied to this cohort which we have recently published [24]*.*

Those patients who died were older, had a longer length of stay and more co-morbidities. A 2-year follow-up period may not be appropriate for this sub-cohort.

A fundamental issue with clinimetric property assessment of summed scores like the PCS is the content overlap [57], as the used subscores are in part textual identical with the summed score, and there also was a high contentual intersection with the XSFMA-F/B and ADL scores. This is difficult to overcome, as the PCS is near ubiquitous in its use for measurement of physical HRQoL. The use of trajectory clustering techniques decreased the risk of bias relative to a researcher-driven approach. The retrospective nature of this analysis mandates that the conclusions are tested prospectively. Trajectory cluster validity is limited by 22 (13.8%) of patients being not classifiable and understanding why these patients have unclear trajectories requires prospective analysis, using a mixed-methods approach. The XSFMA F/B scores have only been validated in German, limiting its use, though it was derived from the English SFMA [62]. Other tools such as the Functional Status Score for the intensive care unit (FSS_ICU) or the physical function in intensive care test scored (PFIT-s) may be of use, having been validated in several countries and languages [35]. While the focus of this manuscript has been on self-reported outcome measures, the subjective nature of these does constitute a limitation and comparative assessment with objective measures in sub-cohorts may be warranted.

### Implications for outcome selection and trial design

As HRQoL outcome measures have often shown a lack of sensitivity in post ICU interventions [54], our data offers two potential methodological solutions: Firstly, the described sub-population characteristics, especially those relating to education could be used as population refinement tools for trials, either as inclusion/exclusion criteria or for differential outcome measures set a priori. This may or may not be feasible where large samples are required, though a differential effect between sub-populations has been used in phase II trials (NCT02358512). Secondly, an adaptive trial design could use (a) the presence of a floor effect as a predictor of a poor trajectory (i.e., a non-responder) in a multi-arm, multi-stage fashion that explores treatments, doses with an option to exclude non-responders [63]; (b) the characteristics (e.g., education or socioeconomic status) for population enrichment that narrow down recruitment to those who are likely to benefit most [64] or (c) the PF score in conjunction with other markers, e.g., CRP (as a marker of persistent inflammation) in a biomarker adaptive design [65] to stratify patients. Lack of data to inform adaptive trial design remains one of the barriers to their use, and this study offers suggestions to overcome this [66].

Both subscore and summary score responsiveness varied over time in both cohorts, with a plateau seen after 6 months. These data imply that physical HRQoL endpoints may be more suited to earlier time points (e.g., 3 and 6 months), and other, more responsive endpoints are needed at 1–2 years such as measures of disability.

## Conclusion

Within sepsis survivors, two distinct recovery trajectories of physical recovery could be demonstrated. Older patient with more co-morbidities and lower educational achievements are more likely to have a trajectory associated with persistent physical impairment. In regard to trajectory prediction, the physical function score of the SF-36 was more responsive than the Physical Component Score of the SF-36 and could be considered for primary outcomes. Future trials should consider adaptive trial designs that can deal with non-responders or sub-cohort specific outcome measures more effectively.

## Supplementary information

**Additional file 1: Additional Table 1.** Categories of Educational Level and Family Status. VT=Vocational Training. GSCE=General Certificate of Secondary Education. **Additional Table 1.1.** Addressed domains of used questionnaires.

**Additional file 2: Additional Table** **2.** Summary measures for Trajectory Projection. eMethods of use of trajectory projection.

**Additional file 3: Additional Table 3.** Baseline characteristics of the whole cohort and the 24 months follow-up cohort. Values shown as medians and interquartile range [IQR] except for ^$^representing mode (range). *P*-values represent two-tailed Mann-Whitney U-tests, except for #=Chi-Squared test. ICULOS= Intensive Care length of stay. MV (d)=period of mechanical ventilation (days), CCI=Charlston Co-morbidity Index, RRT (d)=Renal Replacement Therapy (days), PCS=Physical Component Score of the SF-36, MCS=Mental Component Score recall 3 months prior to critical illness. XSFMA F/B= Extra Short Musculoskeletal Function Assessment regarding Physical Function and Disability, 3m recall=recall 3 months prior to critical illness. NA=Not available, *Categories shown in Additional Table 1. ^1^47 patients without MV, 11 patients without available data, ^2^209 patients without RRT, 5 patients without available data. **Additional Table 3.1.** Baseline characteristics of the whole cohort split by loss to follow-up and death. Values shown as medians and interquartile range [IQR] except for ^$^representing mode (range). ICULOS= Intensive Care length of stay. MV (d)=period of mechanical ventilation (days), CCI=Charlston Co-morbidity Index, RRT (d)=Renal Replacement Therapy (days), PCS=Physical Component Score of the SF-36, MCS =Mental Component Score recall 3 months prior to critical illness. XSFMA F/B= Extra Short Musculoskeletal Function Assessment regarding Physical Function and Disability, 3m recall=recall 3 months prior to critical illness. NA=Not available, *Categories shown in Table S1.

**Additional file 4: Additional Table 4.** Bivariable and multivariate logistic regression analysis of cohort membership characteristics. Dependent variable: Allocation to persistent impairment cohort vs. complete recovery cohort. ICD=International Classification of Disease; ICULOS= Intensive Care Unit Length of Stay. * represents *p*<0.05. **Additional Table 4.1.** Multinomial regression for the persistent impairment group, using the full recovery as the reference group. ICD=International Classification of Disease; ICULOS= Intensive Care Unit Length of Stay; * represents p<0.05. **Additional Table 4.2.** Multinomial regression for the unclustered group, using the full recovery as the reference group. ICD=International Classification of Disease; ICULOS= Intensive Care Unit Length of Stay; * represents p<0.05.

**Additional file 5:** **Additional Table 5.** A and B: Ceiling and floor effects**.** Data are shown as n(%) over time for SF-36 components in patients with a persistent impairment trajectory (*n*=76) and in patients with a completed recovery trajectory (*n*=61) (Table 5A: only patients with completed recovery). PF= Physical Function; RP= Role Physical, BP=Bodily Pain, GH= General Health, XSFMA-F= Extra Short Form Musculoskeletal Function Assessment regarding physical function (F)**.** *represents a value of >15% denoting relevant effect. % may not=100 due to rounding effects.

**Additional file 6: Additional Figure** **1.** Area under receiver operating characteristic curve (AUROC). Logistic regression of predictors of cluster allocation.

**Additional file 7: Additional Figure** **2.** Trajectories of unclustered patients (*n*=22). Data points are means of the SF-36 Physical Component Score (PCS) over 24 months after discharge from ICU.

## Data Availability

The datasets used and/or analysed during the current study are available from the corresponding author on reasonable request.

## Abbreviations

ADL: Activities of daily living
BP: Body pain
GH: General health
HRQoL: Health-related quality of life
ICU: Intensive care unit
PCS: Physical component score
PF: Physical function
PROMS: Patient-reported outcome measures
RP: Role physical
SF-36: Short Form-36 Questionnaire
XSFMA-F/B: Extra Short Form Musculoskeletal Function Assessment regarding physical function (F) and disability (B)
