# Supplementary material for: Implications for post critical illness trial design: sub-phenotyping trajectories of functional recovery among sepsis survivors
Source: Crit Care. 2020 Sep 25;24:577. doi: 10.1186/s13054-020-03275-w (PMC7517819; doi:10.1186/s13054-020-03275-w)
Supplement: Supplementary file 1 — Additional file 1: Additional Table 1. Categories of Educational Level and Family Status. VT=Vocational Training. GSCE=General Certificate of Secondary Education. Additional Table 1.1. Addressed domains of used questionnaires. [file 13054_2020_3275_MOESM1_ESM.docx]

| Category | Educational level | Family Status |
| --- | --- | --- |
| 1 | No degree | Unmarried |
| 2 | Junior high/secondary school, no VT | Married |
| 3 | Junior high/ secondary school, VT | Divorced |
| 4 | GCSE/high school diploma, no VT | Widowed |
| 5 | GCSE/high school diploma, VT | Separated |
| 6 | A level exam/Baccalaureate, no VT | Other |
| 7 | A level exam/Baccalaureate, VT |  |
| 8,9 | University degree |  |

Additional Table 1: Categories of Educational Level and Family Status

VT=Vocational Training

GSCE=General Certificate of Secondary Education

**Additional Table 1.1: Addressed domains of used questionnaires**

| **PF (SF-36)** | **RP (SF-36)** | **BP (SF-36)** | **ADL** | **XSFMA-B** | **XSFMA-F** |
| --- | --- | --- | --- | --- | --- |
| Limitations in… |  | Pain | Difficulties or need for help in… | Affected by problems in… | Difficulties in…. |
| …vigorous activities | Cut down time spent on work | Magnitude | …walking | …house or garden work | …shopping |
| …moderate activities | Accomplished less than would like | Interference with life/work | ..dressing | …bathing, dressing, body care | …climbing stairs |
| …lifting or carrying groceries | Limited in kind of work |  | …bathing | …daily work | …bend over, kneel down |
| …climbing several flights of stairs | Difficulty in performing work |  | …feeding | …stiffness and pain | …dressing |
| …climbing one flight of stairs |  |  | …transfer in/ out bed |  | …using buttons, zippers, press studs or hooks |
| …bending or kneeling |  |  | …using the toilet |  | …walking |
| …walking a mile |  |  | …cooking |  | …walking alone |
| …walking half a mile |  |  | …shopping |  | …hygiene in the toilet |
| …walking one hundred yards |  |  | …making phone calls |  | …turning |
| …bathing or dressing |  |  | …medication intake/ preparation |  | …pursue leisure activities, hobbies, gardening or going out with friends |
|  |  |  | …handling money |  | …light housework or gardening such as dusting, washing dishes or watering flowers |
|  |  |  |  |  | …heavy house or garden work such as mopping the floor, vacuuming and mowing the lawn |
