# Supplementary material for: Implications for post critical illness trial design: sub-phenotyping trajectories of functional recovery among sepsis survivors
Source: Crit Care. 2020 Sep 25;24:577. doi: 10.1186/s13054-020-03275-w (PMC7517819; doi:10.1186/s13054-020-03275-w)
Supplement: Supplementary file 2 — Additional file 2: Additional Table 2. Summary measures for Trajectory Projection. eMethods of use of trajectory projection. [file 13054_2020_3275_MOESM2_ESM.docx]

**Additional Table 2: Summary measures for Trajectory Projection**

1. Range
2. Mean-over-time
3. Standard deviation (SD)
4. Coefficient of variation (CV)
5. Change
6. Mean change per unit time
7. Change relative to the first score
8. Change relative to the mean over time
9. Slope of the linear model
10. R2: Proportion of variance explained by the linear model
11. Maximum of the first differences
12. SD of the first differences
13. SD of the first differences per time unit
14. Mean of the absolute first differences
15. Maximum of the absolute first differences
16. Ratio of the maximum absolute difference to the mean-over-time
17. Ratio of the maximum absolute first difference to the slope
18. Ratio of the SD of the first differences to the slope
19. Mean of the second differences
20. Mean of the absolute second differences
21. Maximum of the absolute second differences
22. Ratio of the maximum absolute second difference to the mean-over-time
23. Ratio of the maximum absolute second difference to mean absolute first difference
24. Ratio of the mean absolute second difference to the mean absolute first difference

**eMethods on use of trajectory projection**

Groups of longitudinal trajectories of Physical Component Scores of the SF-36 were clustered using the R-package TRAJ^1^ and applied. Briefly, this package implemented a 3-step procedure^2^. In the first step, 24 summary measures (available in Additional Table 2) were calculated that describe different features of the trajectories. These measures were chosen as to discriminate between stable–unstable, increasing–decreasing, linear–nonlinear, monotonic–non-monotonic patterns of change^2^.

In the second step, any highly correlated measures were removed (change and mean change over time; standard deviation of the first differences and standard deviation of the first differences per time unit; indicated in Additional Table 2 above) and the remainder was then analysed using factor analysis. Factor analysis was applied to the matrix to choose a subset of non-redundant measures that best describe the main features of trajectories.

In the third step, kmeans was used to cluster the trajectories represented by vectors of these non-redundant measures. As kmeans randomly selects data points as cluster centroids, it is usually recommended to repeat the analysis several times with different random seeds and to select the best configuration of the different trials as the final clustering. Here, we used kmeans with 50 random seeds. The optimal number of clusters was determined using the elbow method: The data was clustered into 2-10 clusters and the sum of square distances from each point to its assigned center was computed. The average distance was plotted against the number of clusters. The best number of clusters was determined by the position of an “elbow” (the point of inflection) of the line plot.

Based on this method we chose to cluster the trajectories into 10 clusters. Of these 10 clusters, we observed two main clusters with distinct trajectories reflecting clinically meaningful recovery, while the trajectory patterns of the remaining clusters were less defined. Based on these observations, we chose to keep the two distinct trajectory clusters and to group the remaining trajectories into one cluster yielding three different clusters for all downstream analyses.

References:

1. https://cran.r-project.org/web/packages/traj/ Accessed 06 Mar 2020.

2. Leffondre K, Abrahamowicz M, Regeasse A, et al. Statistical measures were proposed for identifying longitudinal patterns of change in quantitative health indicators. J Clin Epidemiol. 2004;57(10):1049-1062.
