# Supplementary material for: Implications for post critical illness trial design: sub-phenotyping trajectories of functional recovery among sepsis survivors
Source: Crit Care. 2020 Sep 25;24:577. doi: 10.1186/s13054-020-03275-w (PMC7517819; doi:10.1186/s13054-020-03275-w)
Supplement: Supplementary file 3 — Additional file 3: Additional Table 3. Baseline characteristics of the whole cohort and the 24 months follow-up cohort. Values shown as medians and interquartile range [IQR] except for $representing mode (range). P-values represent two-tailed Mann-Whitney U-tests, except for #=Chi-Squared test. ICULOS= Intensive Care length of stay. MV (d)=period of mechanical ventilation (days), CCI=Charlston Co-morbidity Index, RRT (d)=Renal Replacement Therapy (days), PCS=Physical Component Score of the SF-36, MCS=Mental Component Score recall 3 months prior to critical illness. XSFMA F/B= Extra Short Musculoskeletal Function Assessment regarding Physical Function and Disability, 3m recall=recall 3 months prior to critical illness. NA=Not available, *Categories shown in Additional Table 1. 147 patients without MV, 11 patients without available data, 2209 patients without RRT, 5 patients without available data. Additional Table 3.1. Baseline characteristics of the whole cohort split by loss to follow-up and death. Values shown as medians and interquartile range [IQR] except for $representing mode (range). ICULOS= Intensive Care length of stay. MV (d)=period of mechanical ventilation (days), CCI=Charlston Co-morbidity Index, RRT (d)=Renal Replacement Therapy (days), PCS=Physical Component Score of the SF-36, MCS =Mental Component Score recall 3 months prior to critical illness. XSFMA F/B= Extra Short Musculoskeletal Function Assessment regarding Physical Function and Disability, 3m recall=recall 3 months prior to critical illness. NA=Not available, *Categories shown in Table S1. [file 13054_2020_3275_MOESM3_ESM.docx]

|  | All | NA | Follow-up cohort | NA |  |
| --- | --- | --- | --- | --- | --- |
| n | 291 |  | 159 |  |  |
| Age | 64.0 (52.8-73) |  | 61 (51-71) |  | P=0.178 |
| Male Sex^#^ | 192 (66.2%) |  | 107 (67.3%) |  | P=0.931 |
| ICULOS (d) | 26 (12.8-46.0) | 27 | 23.0 (12-40) | 10 | P=0.058 |
| MV(d) | 8 (4-15) | 58^[[1]](#footnote-1)^ | 8.0 (2-22) | 6 | P=0.323 |
| CCI | 4 (2-6) | 2 | 3 (1-5) | 1 | P=0.050 |
| RRT (d) | 0 (0-2) | 214^[[2]](#footnote-2)^ | 0 (0-2) | 4 | P=0.804 |
| Tracheostomy^#^ | 97 (43.3%) | 72 | 49 (30.8%) | 37 | P=0.230 |
| Intervention group^#^ | 148 (51%) |  | 87 (55%) |  | P=0.456 |
| Family status*^$^ | 2 (1-6) | 6 | 2 (1-6) | 2 | P=0.417 |
| Education Level*^$^ | 5(1-9) | 2 | 5 (1-9) |  | P=0.415 |
| BMI at discharge | 27.1 (23.3-30) | 12 | 27 (23.2-30.5) | 4 | P=0.890 |
| PCS | 24 (19-31) | 27 | 24.8 (20-31.3) |  | P=0.351 |
| MCS | 50 (38-60) | 27 | 48.2 (40-58.9) |  | P=0.881 |
| PCS- 3m recall | 45 (33-55) | 24 | 46 (36-56) |  | P=0.387 |
| MCS-3m recall | 56 (48-60) | 10 | 55.7 (48.5-60.4) |  | P=0.761 |
| XSFMA-F 3m recall | 2 (0-25) | 75 | 0 (0-19.7) | 2 | P=0.228 |
| XSFMA-B 3m recall | 3 (0-25) | 75 | 0 (0-19) | 2 | P=0.361 |
| No. of ICD diagnoses  at discharge | 9 (6-14) | 13 | 9 (6-14) |  | P=0.794 |

**Additional Table 3: Baseline characteristics of the whole cohort and the 24 months follow-up cohort**

Values shown as medians and interquartile range [IQR] except for ^$^representing mode (range). P-values represent two-tailed Mann-Whitney U-tests, except for #=Chi-Squared test. ICULOS= Intensive Care length of stay. MV (d)=period of mechanical ventilation (days), CCI=Charlston Co-morbidity Index, RRT(d)=Renal Replacement Therapy (days), PCS=Physical Component Score of the SF-36, MCS =Mental Component Score recall 3 months prior to critical illness. XSFMA F/B= Extra Short Musculoskeletal Function Assessment regarding Physical Function and Disability, 3m recall=recall 3 months prior to critical illness. NA=Not available, *Categories shown in Table S1

|  | All | Follow-up cohort | Died | Lost to follow up |
| --- | --- | --- | --- | --- |
| n | 291 | 159 | 90 | 42 |
| Age (y) | 64.0 (52.8-73) | 61 (51-71) | 68 (59-73) | 63.5 (48.5-73) |
| Male Sex | 192 (66.2%) | 107 (67.3%) | 61 (67.7%) | 26 (61.9%) |
| ICULOS (d) | 26 (13-46) | 23.0 (12-40) | 34(15-57) | 26 (11-42) |
| MV(d) | 8 (4-15) | 8.0 (2-22) | 10 (1-30) | 6 (1-23) |
| CCI | 4 (2-6) | 3 (1-5) | 5 (2-7) | 4 (2-7) |
| RRT (d) | 0 (0-2) | 0 (0-2) | 0 (0-3) | 0 (0-0) |
| Tracheostomy | 97 (43.3%) | 49 (30.8%) | 33 (36.7%) | 15(35.7%) |
| Intervention group | 148 (51%) | 87 (55%) | 47 (52.2%) | 24 (57.1%) |
| Family status*^$^ | 2 (1-6) | 2 (1-6) | 2 (1-6) | 2 (1-6) |
| Education Level*^$^ | 5(1-9) | 5 (1-9) | 5 (1-9) | 5 (2-6) |
| BMI at discharge | 27.1 (23.3-30) | 27 (23.2-30.5) | 26.4 (34.4-29.7) | 27.7 (23-32) |
| PCS^a^ | 24 (19-31) | 24.8 (20-31.3) | 22.5(18.0-30.5) | 23.5 (17-29.8) |
| MCS^a^ | 50 (38-60) | 48.2 (40-58.9) | 52 (36.3-61.0) | 52.5 (38-62) |
| PCS- 3m recall^a^ | 45 (33-55) | 46 (36-56) | 39 (27-52) | 50 (35-55.5) |
| MCS-3m recall^a^ | 56 (48-60) | 55.7 (48.5-60.4) | 76 (68-84.5) | 76 (56-88) |
| XSFMA-F 3m recall^b^ | 2 (0-25) | 0 (0-19.7) | 24 (6.5-43.5) | 18 (5.5-44.3) |
| XSFMA-B 3m recall^b^ | 3 (0-25) | 0 (0-19) | 44 (22-72) | 38 (19-62.5) |
| No. of ICD diagnoses  at discharge | 9 (6-14) | 9 (6-14) | 10 (6-14) | 8 (4.8-13.3) |

**Additional Table 3.1: Baseline characteristics of the whole cohort and the 24 months follow-up cohort**

Values shown as medians and interquartile range [IQR] except for ^$^representing mode (range). ICULOS= Intensive Care length of stay. MV (d)=period of mechanical ventilation (days), CCI=Charlston Co-morbidity Index, RRT(d)=Renal Replacement Therapy (days), PCS=Physical Component Score of the SF-36, MCS =Mental Component Score recall 3 months prior to critical illness. XSFMA F/B= Extra Short Musculoskeletal Function Assessment regarding Physical Function and Disability, 3m recall=recall 3 months prior to critical illness. NA=Not available, *Categories shown in Table S1.

^a^ Range of possible scores, 0-100. High score indicates low impairment. ^b^ Range of possible scores, 0-100. High score indicates high impairment.

1. 47 patients without MV, 11 patients without available data [↑](#footnote-ref-1)
2. 209 patients without RRT, 5 patients without available data [↑](#footnote-ref-2)
