# Supplementary material for: Implications for post critical illness trial design: sub-phenotyping trajectories of functional recovery among sepsis survivors
Source: Crit Care. 2020 Sep 25;24:577. doi: 10.1186/s13054-020-03275-w (PMC7517819; doi:10.1186/s13054-020-03275-w)
Supplement: Supplementary file 4 — Additional file 4: Additional Table 4. Bivariable and multivariate logistic regression analysis of cohort membership characteristics. Dependent variable: Allocation to persistent impairment cohort vs. complete recovery cohort. ICD=International Classification of Disease; ICULOS= Intensive Care Unit Length of Stay. * represents p<0.05. Additional Table 4.1. Multinomial regression for the persistent impairment group, using the full recovery as the reference group. ICD=International Classification of Disease; ICULOS= Intensive Care Unit Length of Stay; * represents p<0.05. Additional Table 4.2. Multinomial regression for the unclustered group, using the full recovery as the reference group. ICD=International Classification of Disease; ICULOS= Intensive Care Unit Length of Stay; * represents p<0.05. [file 13054_2020_3275_MOESM4_ESM.docx]

**Additional Table 4: Bivariable and multivariate logistic regression analysis of cohort membership characteristics**

|  | bivariable logistic regression | | | multivariate logistic regression | | |
| --- | --- | --- | --- | --- | --- | --- |
| Variable | OR | 95%CI | P value | OR | 95%CI | P value |
| Age | 1.042 | 1.016-1.069 | 0.002 | 1.034 | 1.006- 1.064 | **0.019*** |
| ICULOS | 1.010 | 0.996-1.025 | 0.162 | 1.010 | 0.995- 1.027 | 0.186 |
| Education level | 0.823 | 0.701-0.967 | 0.018 | 0.829 | 0.692- 0.984 | **0.035*** |
| BMI at discharge | 1.076 | 1.017-1.139 | 0.012 | 1.058 | 1.001- 1.127 | 0.059 |
| Family Status | 0.742 | 0.527-1.015 | 0.072 | 1.263 | 0.889- 1.828 | 0.198 |
| No of ICD diagnoses  at discharge | 1.072 | 1.001-1.148 | 0.048 | 1.081 | 1.003- 1.170 | **0.046*** |

Dependent variable: Allocation to persistent impairment cohort vs. complete recovery cohort. ICD=International Classification of Disease; ICULOS= Intensive Care Unit Length of Stay; * represents p<0.05

**Additional Table 4.1: Multinomial regression for the persistent impairment group, using the full recovery as the reference group**

| Variable | OR | 95%CI | P value |
| --- | --- | --- | --- |
| Age | 1.042 | 1.010-1.074 | **0.008*** |
| ICULOS | 1.010 | 0.993-1.027 | 0.243 |
| Education level | 0.823 | 0.685-0.988 | **0.037*** |
| BMI at discharge | 1.082 | 0.999-1.171 | 0.051 |
| Family Status | 1.087 | 1.010-1.171 | **0.027*** |
| No of ICD diagnoses | 1.217 | 0.834-1.775 | 0.309 |

ICD=International Classification of Disease; ICULOS= Intensive Care Unit Length of Stay; * represents p<0.05

**Additional Table 4.2: Multinomial regression for the unclustered group, using the full recovery as the reference group.**

| Variable | OR | 95%CI | P value |
| --- | --- | --- | --- |
| Age | 1.032 | 0.991-1.075 | 0.130 |
| ICULOS | 1.013 | 0.992-1.035 | 0.218 |
| Education level | 0.911 | 0.711-1.168 | 0.462 |
| No of ICD diagnoses | 1.075 | 0.967-1.196 | 0.181 |
| BMI at discharge | 1.029 | 0.931-1.138 | 0.574 |
| Family Status | 0.989 | 0.563-1.734 | 0.968 |

ICD=International Classification of Disease; ICULOS= Intensive Care Unit Length of Stay; * represents p<0.05
