# Supplementary material for: Implications for post critical illness trial design: sub-phenotyping trajectories of functional recovery among sepsis survivors
Source: Crit Care. 2020 Sep 25;24:577. doi: 10.1186/s13054-020-03275-w (PMC7517819; doi:10.1186/s13054-020-03275-w)
Supplement: Supplementary file 5 — Additional file 5: Additional Table 5. A and B: Ceiling and floor effects. Data are shown as n(%) over time for SF-36 components in patients with a persistent impairment trajectory (n=76) and in patients with a completed recovery trajectory (n=61) (Table 5A: only patients with completed recovery). PF= Physical Function; RP= Role Physical, BP=Bodily Pain, GH= General Health, XSFMA-F= Extra Short Form Musculoskeletal Function Assessment regarding physical function (F). *represents a value of >15% denoting relevant effect. % may not=100 due to rounding effects. [file 13054_2020_3275_MOESM5_ESM.docx]

**Additional Table 5A: Ceiling effects**

| Months | 0 | 6 | 12 | 24 |
| --- | --- | --- | --- | --- |
| PF | 3 (5) | 5 (8) | **10 (16)*** | **9 (15)*** |
| RP | 4 (7) | **16 (26)*** | **26 (43)*** | **27 (44)*** |
| BP | **28(46)*** | **37(61)*** | **37(61)*** | **35(57)*** |
| GH | 0 (0) | 0 (0) | 0 (0) | 0 (0) |
| XSFMA-F | N/A | 0 (0) | 0 (0) | 0 (0) |

**Additional Table 5B: Floor effects**

| Months | 0 | 6 | 12 | 24 |
| --- | --- | --- | --- | --- |
| Persistent impairment | | | | |
| PF | **50 (66)*** | 11(14) | 9 (12) | **16 (21)*** |
| RP | **67 (88)*** | **55 (72)*** | **53 (70)*** | **54 (71)*** |
| BP | **10 (17)*** | 5 (8) | 2 (2.6) | 7(12) |
| GH | 0 (0) | 0 (0) | 0 (0) | 0 (0) |
| XSFMA-F | **N/A** | 0 (0) | 0 (0) | 0 (0) |
| Completed recovery | | | | |
| PF | **19(31)*** | 0 (0) | 0 (0) | 0 (0) |
| RP | **48(79)*** | **18(30)*** | 8(13.1) | **9(15)*** |
| BP | 3 (3.3) | 0 (0) | 0 (0) | 2(2) |
| GH | 0(0) | 0 (0) | 0 (0) | 0 (0) |
| XSFMA-F | N/A | **15(20)*** | **26(34)*** | **35(46)*** |

Data are shown as n (%) over time for SF-36 components in patients with a persistent impairment trajectory (n=76) and in patients with a completed recovery trajectory (n=61) (Table 5A: only patients with completed recovery). PF= Physical Function; RP= Role Physical, BP=Bodily Pain, GH= General Health, XSFMA-F= Extra Short Form Musculoskeletal Function Assessment regarding physical function (F). *Represents a value of >15% denoting relevant effect. % may not=100 due to rounding effects.
