# Supplementary figures and images for: Implications for post critical illness trial design: sub-phenotyping trajectories of functional recovery among sepsis survivors
Source: Crit Care. 2020 Sep 25;24:577. doi: 10.1186/s13054-020-03275-w (PMC7517819; doi:10.1186/s13054-020-03275-w)

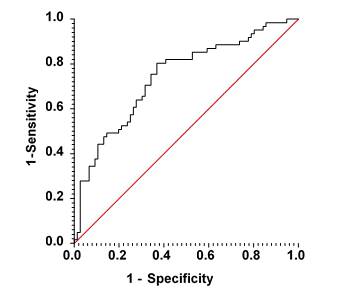

Supplement: Supplementary file 6 — Additional file 6: Additional Figure 1. Area under receiver operating characteristic curve (AUROC). Logistic regression of predictors of cluster allocation. [file 13054_2020_3275_MOESM6_ESM.jpg]

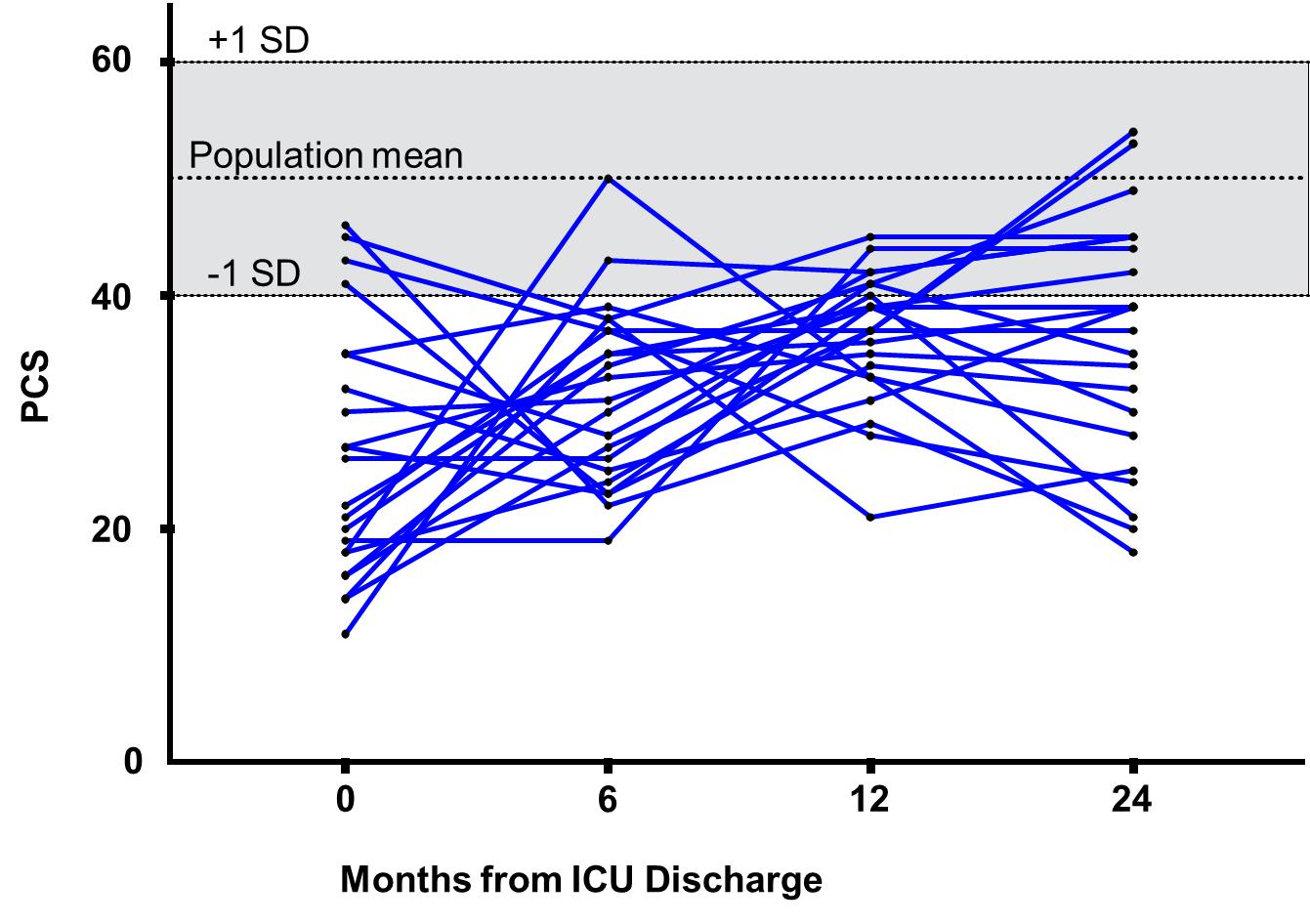

Supplement: Supplementary file 7 — Additional file 7: Additional Figure 2. Trajectories of unclustered patients (n=22). Data points are means of the SF-36 Physical Component Score (PCS) over 24 months after discharge from ICU. [file 13054_2020_3275_MOESM7_ESM.jpg]
